# The New Building of the Baltimore College of Dental Surgery

**Published:** 1881-06

**Authors:** 


### EDITORIAL, ETC.

---

*The New Building of the Baltimore College of Dental Surgery.*—During the present month, June, this Institution occupies its new building S. E. Cor. of Eutaw and Franklin Sts., some three squares north of its former location.

The increase in the number of students for several years past, rendered the old building, commodious as it was, too contracted, and hence a change became absolutely necessary.

The result is the New College Building, a large and handsome structure, fronting on two wide streets, built of the finest pressed brick, with elaborate granite facings and ornaments, and which is, beyond doubt, the most complete and handsome building devoted to dental education in the world.

The College occupies the entire structure with the exception of the large stores on the ground floor.

The "Infirmary Hall," located on the *second story,* is lighted by a large number of windows on three sides, extending from near the floor to the ceiling, and is a model of architectural beauty, besides being handsomely furnished. Crimson lambrequins with gilt cornices, and beautiful and costly chandeliers, which latter are found in every room, adorn the windows and walls. Of a size capable of accomodating hundreds of patients, this handsome and spacious "Infirmary Hall," is perfect in its adaptation to the practical work of Operative Dentistry, and it can be truthfully said that it is unequalled anywhere.

On the same floor, opposite to the Infirmary, is the large and handsome Hall devoted to the extensive "Museum" of the College, which for number and value of dental pathological specimens, accumulating for more than forty years, cannot be excelled by any other institution. In the rear of the Museum is a handsome room devoted to the extraction of teeth, which is sufficiently distant from the Infirmary to prevent any noise made by patients undergoing such an operation, being heard in the latter Hall.

On this floor are also a number of water closets, stationary marble washstands, hat and coat room, and every other convenience.

On the third floor, and fronting on two streets, like Infirmary Hall, is the large "Lecture Hall," capable of comfortably seating over *three hundred* students, with a large number of high windows, and adorned with centre pieces, beautiful chandeliers and cornices.

Opposite the Lecture Hall, are *three* rooms devoted to "Laboratory" purposes, two of which are large Halls, and the third a separate room devoted exclusively to plaster and furnace work, vulcanizing, &c The main entrance is on Eutaw Street, wide and handsome stairways leading to the different Halls. The entire building is heated by steam, with gas and water in every room. The Dissecting Room is situated in the upper story, and is capable of accommodating a number of classes engaged in the study of practical anatomy.

Devoted wholly to dental purposes, and unlike some other institutions connected with Medical Colleges, which make a considerable display of buildings wherein they are compelled to occupy the basement rooms only, while the more pleasant and acceptable ones are assigned to the Medical department, we can truthfully assert that the Baltimore College of Dental Surgery now occupies the most complete building in America devoted to dental education.

